# BATS: Adaptive Ultra Low Power Sensor Network for Animal Tracking

**DOI:** 10.3390/s18103343

**Published:** 2018-10-07

**Authors:** Niklas Duda, Thorsten Nowak, Markus Hartmann, Michael Schadhauser, Björn Cassens, Peter Wägemann, Muhammad Nabeel, Simon Ripperger, Sebastian Herbst, Klaus Meyer-Wegener, Frieder Mayer, Falko Dressler, Wolfgang Schröder-Preikschat, Rüdiger Kapitza, Jörg Robert, Jörn Thielecke, Robert Weigel, Alexander Kölpin

**Affiliations:** 1 Institute for Electronics Engineering, Friedrich-Alexander University of Erlangen-Nürnberg, 91058 Erlangen, Germany; weigel@lte.e-technik.uni-erlangen.de; 2 Institute of Information Technology, Friedrich-Alexander University of Erlangen-Nürnberg, 91058 Erlangen, Germany; thorsten.nowak@fau.de (T.N.); markus.hartmann@fau.de (M.H.); michael.schadhauser@fau.de (M.S.); joerg.robert@fau.de (J.R.); joern.thielecke@fau.de (J.T.); 3 Institute of Operating Systems and Computer Networks, TU Braunschweig, 38106 Brunswick, Germany; cassens@ibr.cs.tu-bs.de (B.C.); kapitza@ibr.cs.tu-bs.de (R.K.); 4 Chair of Computer Science 4—Distributed Systems and Operating Systems, Friedrich-Alexander University of Erlangen-Nürnberg, 91058 Erlangen, Germany; waegemann@cs.fau.de (P.W.); wosch@cs.fau.de (W.S.P.); 5 Heinz Nixdorf Institute and Department of Computer Science, Paderborn University, 33098 Paderborn, Germany; nabeel@ccs-labs.org (M.N.); dressler@ccs-labs.org (F.D.); 6 Museum für Naturkunde, Leibniz-Institute for Evolution and Biodiversity Science, 10115 Berlin, Germany; simon.ripperger@mfn-berlin.de (S.R.); frieder.mayer@mfn.berlin (F.M.); 7 Smithsonian Tropical Research Institute, Apartado 0843-03092, Balboa, Panama; 8 Chair of Computer Science 6—Data Management, Friedrich-Alexander University of Erlangen-Nürnberg, 91058 Erlangen, Germany; sebastian.herbst@fau.de (S.H.); klaus.meyer-wegener@fau.de (K.M.W.); 9 Berlin-Brandenburg Institute of Advanced Biodiversity Research (BBIB), 14195 Berlin, Germany; 10 Chair for Electronics and Sensor Systems, Brandenburg University of Technology, 03046 Cottbus, Germany; alexander.koelpin@b-tu.de

**Keywords:** wireless sensor networks, animal tracking, adaptive sensor network

## Abstract

In this paper, the BATS project is presented, which aims to track the behavior of bats via an ultra-low power wireless sensor network. An overview about the whole project and its parts like sensor node design, tracking grid and software infrastructure is given and the evaluation of the project is shown. The BATS project includes a lightweight sensor node that is attached to bats and combines multiple features. Communication among sensor nodes allows tracking of bat encounters. Flight trajectories of individual tagged bats can be recorded at high spatial and temporal resolution by a ground node grid. To increase the communication range, the BATS project implemented a long-range telemetry system to still receive sensor data outside the standard ground node network. The whole system is designed with the common goal of ultra-low energy consumption while still maintaining optimal measurement results. To this end, the system is designed in a flexible way and is able to adapt its functionality according to the current situation. In this way, it uses the energy available on the sensor node as efficient as possible.

## 1. Introduction

Biologging or the remote tracking of animals by means of attached tags is motivating interdisciplinary research for more than 50 years and has been strongly technology-driven ever since. While biologists have mainly focused on individual movement patterns during the first decades of biologging research, applications have become much broader recently by the availability of digital transceivers and manifold sensors for designing animal borne tags. Advances in biologging technology have motivated innovative research in the fields of movement ecology, sociobiology or conservation biology, which relies on detailed information on the behavior of individual animals [1,2,3]. For example, biologging studies have revealed how movement rates of mammals respond to anthropogenic impact [4] or how social groups of primates make decision on where to move [5].

Advanced biologging devices such as Global Positioning System (GPS) -tags automatically collect data and enable remote download, which allows for the observation of large numbers of individuals at a time, maximizes data recovery rates and minimizes the impact on study objects. Generated datasets become increasingly rich since integrated sensors collect precise information on physiological or environmental conditions in addition to the location of the tagged animals. However, researchers always face the trade-off between performance and device weight since more complex functionalities come at the cost of higher energy expense, which translates into higher tag weight by the demand for a bigger battery. Energy harvesting solutions, such as solar cells, recharge the battery while the animal is on the move and extend battery life. However, applications are mainly restricted to diurnal taxa which range in sun-exposed environments and its recharging efficiency may depend upon weather or seasonal conditions [6]. In turn, tag weight dictates the spectrum of animal species which can be studied because tags constitute a burden to the animal, which may induce unintended changes in behavior or even reduce fitness or survival rates [7]. Considerations for tag to body weight ratio vary across taxa suggesting tags not to exceed 3–5% or for short-term studies a maximum of 10% of the body weight [8,9,10,11]. Due to these limitations and the considerably large size and weight of most fully automated tracking devices, around two thirds of avian and mammalian species still cannot be studied [2]. For this reason, older techniques which originated between the 1960s and 1990s, which rely on smaller but less powerful animal-borne devices such as VHF-transmitters, geolocators or Passive Integrated Transponder (PIT) tags still represent the state-of-the-art for studying smaller vertebrates like songbirds, rodents or bats.

The most diverse part of the mammalian and avian range of species is at a body mass of around 10–20 g [2] and can therefore only be studied with tags that weigh 2 g or less. Hence, the further miniaturization of biologging technologies, which are capable of fully automated tracking of miniaturized tags at high spatial and temporal resolution, collecting complementary sensor data and remote access for download and reconfiguration would represent a quantum-leap for biologging research. Wireless sensor networks (WSNs) may be an ideal solution to meet the aforementioned criteria if hardware, software and communication protocols are designed with the goal of ultra-low-power consumption in order to ensure an acceptable runtime of at least 1–2 weeks. We present an adaptive and reconfigurable ultra-low-power WSN for biologging that enables ground-based localization at high temporal and spatial resolution, communication among animal-borne tags for direct encounter detection and remote data access, optionally via long-range telemetry. We verify our developments by tracking free-ranging bats, an animal group which is particularly difficult to observe due to its nocturnal activity, high mobility and small body weight of most species.

## 2. Related Work

Most available advanced animal tracking systems rely on relatively big mobile nodes. These of course comprise broader functionality; however, they cannot be used for tracking small-bodied animals like most bat species. Here, the strict size and weight constraints limit the scope of suitable tracking methods. In the past, a common approach was to use Very High Frequency (VHF)-Tags that periodically send a modulated signal that can be used to track the animal. Due to the nature of these tags, it requires a lot of manual work to track the animals and the number of track-able individuals at the same time is limited. In addition, these tags do not provide any additional information about the individual besides a rough geographic position.

In the following, some advanced systems that allow automated tracking of multiple individuals and are focusing on small-bodied species are shortly described.

### 2.1. Ground Based Tracking

The **ATLAS** [12] system (Advanced Tracking and Localization of Animals in real-life Systems) is based on a time-of-arrival principle for reliable localization of digital transceivers, which weigh around 1 g, over distances of up to 15 km. The initial real-world deployment of the ATLAS system consisted of nine base stations covering an area of several km${}^{2}$ in the Hula Valley in Israel. The runtime of the nodes is highly weight dependent. A 1.5 g heavy tag can reach a runtime of 10 days. A 10 g heavy node already reached a runtime of 100 days. The system can track the transceivers with a standard deviation of 5 m, which is comparable in terms of accuracy to GPS-tracking devices and allows studying space use at a high spatial resolution.

The **MOTUS** wildlife tracking system [13] benefits from the lightweight quality of traditional VHF tags by tracking animals over large geographic scales in a collaborative approach. Arrays of automated receivers, which are curated by different research groups, detect digital radio-telemetry transmitters emitting signals at a single frequency. This way all participants obtain data from collaboratively maintained infrastructure. The VHF-tags used in the MOTUS project, which are relatively simple in terms of functionality, weigh 0.2 to 2.6 g at lifetimes of 10 days to three years. The tags send out a unique signal, respectively, and that way a unique ID can be assigned to each tag. The system supports up to 500 unique IDs. The reception range of the used base stations is between 500 m to 15 km depending on the antenna setup. The location of the tag gets derived from which station receives the signal. Thus, while the system covers a large geographic scale, the resolution in this area is limited, making the setup an ideal instrument for tracking large-scale movement of animals.

**Encounternet** [14] is an approach for automated encounter logging to study social behavior in wild animals. Other than previous encounter tracking implementations, it does not require retrieving the sensor nodes but includes ground stations that are downloading the recorded data from the mobile nodes. The Encounternet tags have a runtime of about 7.5 days when used only as transmitters and 21 hours in real proximity logging mode with transmitting and receiving enabled while having a weight of 1.3 g. This allows the encounter tracking of lightweight species but has highly limited runtime and does not include absolute location tracking. Previous versions of the Encounternet sensor nodes still had a weight of 10 g and have been used for proximity logging over a time period of ca. two months in a single deployment [15].

### 2.2. Satellite Based Tracking

Traditional **GPS trackers** are widely used for animal tracking. These contain a GPS module that is periodically woken up to perform a GPS fix to record its location. Depending on the system, it is required to recover the tags to download the recorded fixes from the internal memory or the tag contains a mobile network modem to automatically upload the data. The smallest versions of simple loggers can be as light as 1 g, but they can only record around 100 fixes over several days. If long-term high-resolution tracking and an option for remote download are required, tag weight rapidly increases [2]. While these trackers reach high spatial resolution in free space (like tracking migrating birds while flying), data quality may suffer when animals are located in places with poor GPS reception such as thick forests or inside roosts (e.g., cavern or tree holes).

The **ICARUS** project [16] (International Cooperation for animal Research Using Space) has the goal to enable tracking of small objects such as migratory bats or birds. This is achieved via an antenna attached to the International Space Station (ISS), which receives data from animal-borne tags. The mobile tags document their location via GPS and contain accelerometer, magnetometer and temperature sensors. Whenever the ISS is in range, the recorded positions and sensor data will be uploaded to the ISS and from there stored in a database. The big advantage of the ICARUS project is the relatively low orbit of the ISS. Thus, uploading of data can be realized even with low energy and therefore tags can be smaller than conventional GPS trackers with remote access. The ISS uplink of the ICARUS project allows nearly global coverage. The current state of the project uses sensor tags with a weight of 5 g and volume of 2 cm${}^{2}$. Thanks to supporting solar cells, the runtime can be extended over the one supported by the battery capacity as long as diurnal species are investigated. There are plans to further reduce the weight of the sensor nodes by reducing the functionality to support tracking of even smaller animals.

## 3. BATS System Overview

**Figure 1:**
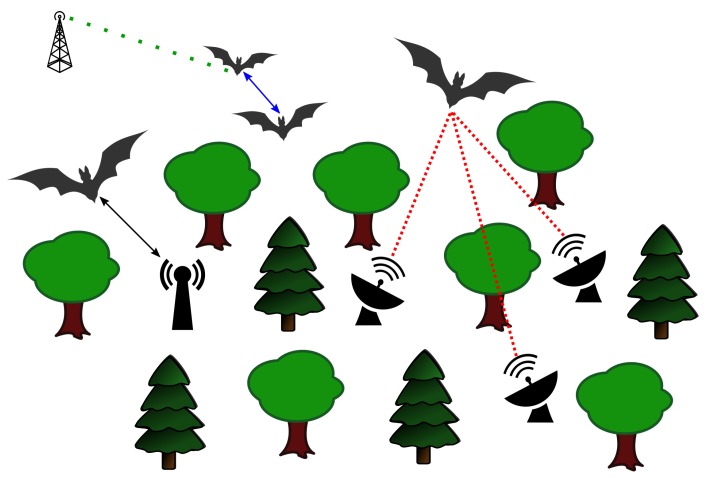
Conceptional overview about the abilities of the BATS system: Trajectory tracking (red dotted line), Encounter Detection (node-to-node communication in solid blue and download in solid black) and Long Range Telemetry (dotted green).

The BATS Project (http://www.for-bats.org/) aims to solve the challenges described in Section 1. The project is split into multiple sub-projects, each working on specific details of the whole system. An overview of the application of the BATS project is given in Figure 1. The system allows a so called encounter detection, meaning that the sensor tags can notice that another tagged bat is close and record the duration of the meeting as well as estimate a rough distance based on Received Signal Strength Indicator (RSSI) values (blue solid line). The encounter detection works independent of any ground station network and can record the encounter data until a base station is in range again and downloads the data (black solid line). While being in the ground station tracking network, in addition to simple encounter detection, the BATS system also allows the localization of the bats in reference to the ground network and the recording of flight trajectory. This is achieved by calculating an expected position of the bat based on the signal received by multiple base stations (red tightly dotted line). To be less dependent on the bat staying in the ground network, we also implemented a long range telemetry system that can receive data of the bats over a high range (green light dotted line). The long range, however, results in a limited data rate. This makes it impossible to transmit the same data as in the ground node network but still we are able to get some information about the investigated individuals.

The implementation of all functions of the BATS project focuses on minimizing the energy consumption of the mobile node. Systems for node-to-node communication like Bluetooth 5 [17] and long range telemetry like LoRa are already available. However, using them is not feasible here since it would require implementing multiple protocols with their corresponding overhead in order to realize the complete set of functionalities of the BATS system. Even though Bluetooth 5 allows efficient energy management for wireless sensor networks [18], we would not consider Bluetooth or other already existing systems a suitable solution for node-to-node communication in the BATS project since the research object bat poses a set of new challenges compared to industrial sensor networks. This is mostly due to the behavior and habitat of bats as well as the strict weight and size limit. By implementing our own radio protocols, we can keep the overhead low and allow quick switching between the different operating modes.

The following chapters will give an insight about how the single parts of the BATS system are built. The mobile sensor node with the embedded software is the central part of the sensor network and gets attached to the bats. Depending on the desired application mode and the location of the bat, a different set of ground network nodes communicates with the sensor tag on the bat for encounter detection data download, localization or long range telemetry. As a final step, we developed methods to sort the vast amount of collected data to allow researches to investigate the biologic questions this system was developed to answer.

### 3.1. Mobile Node

#### 3.1.1. Overall Functionality

**Figure 2:**
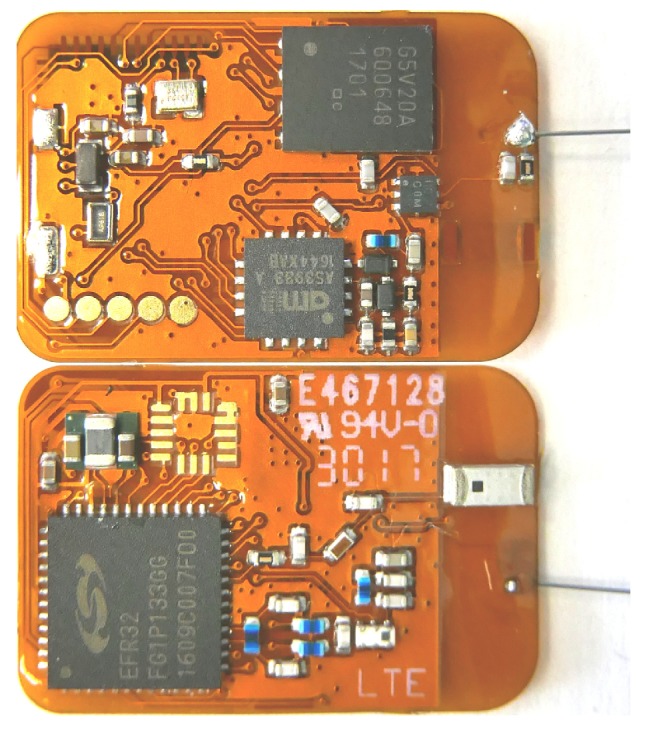
Front and back of mobile node.

For the monitoring of the activity of bats, these have to be tagged with a wireless sensor node. The current version of the BATS sensor node is shown in Figure 2. Attached to the bat, the mobile node serves multiple functions which are depending on the current location of the bat. We introduced so called zones that change the actual behavior of the tag. If in the range of the ground tracking grid (Section 3.3), two beacons are sent out at 868/915 MHz and 2.4 GHz eight times per second. Otherwise, these beacons are omitted to save energy. The current zone respectively operating mode is set by beacons sent from a ground station. In the current implementation, the encounter detection is active regardless of the current location and beacons are periodically sent out for other mobile nodes to receive.

#### 3.1.2. Hardware Setup

**Figure 3:**
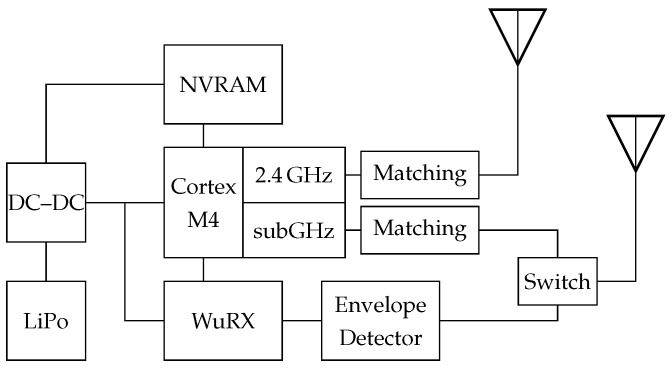
Overview of mobile node design.

An overview about the architecture of the BATS mobile node is shown in Figure 3. A Silabs EFR32 Flex Gecko System-on-Chip (SoC) (Austin, TX, USA) is the central component in the design. It combines a Cortex M4 processor core with two radio frontends, one for the 2.4 GHz Industrial, Scientific and Medical (ISM)Band and one subGHz transceiver. Depending on the desired location for the sensor network, the frequency of the subGHz transceiver is set to the 868 MHz (Europe) or 915 MHz (America) band. The wake-up functionality is implemented with an AMS AS3933 wake-up receiver (Unterpremstätten, Austria) put behind an envelope detector. The envelope detector is used to extract the low frequency wake-up pattern from the high frequency radio signal. To increase the data storage capacity, a Ferroelectric Random Access Memory (FRAM) is used as Non-Volatile Random Access Memory (NVRAM). The advantages of FRAM compared to standard flash memory are the highly reduced power consumption and fast read and write access. A detailed description of the selected hardware can be found in Ref. [19].

The system can easily be expanded with additional functionality like new sensors. An accelerometer could be used for activity detection of bats and, in combination with a magnetometer and gyroscope, the inclination and heading direction could be calculated. However, the focus in the proposed design was on a ultra lightweight and ultra low power system. Thus, no additional sensors are used in the current version of the BATS mobile node.

#### 3.1.3. Wake-Up Receiver Based Communication Approach

The key part of the proximity sensing is the node-to-node communication. Especially with a high number of supported nodes, it is not feasible anymore to turn on the radio in predefined time slot to receive potential packages. Instead, it would be necessary to constantly listen to incoming packages. To still be able to keep the power consumption to a minimum, the BATS mobile node makes use of a wake-up receiver based approach. This ultra low power receiver is constantly in receiving mode and wakes up the remaining circuit upon reception of a special beacon. The downside of the low power consumption is the relatively low sensitivity of −43 dBm and thus a limited range of a few meters. For proximity logging, however, this is the preferred behavior. This way, only close encounters will activate the system and it is not necessary to implement a software RSSI threshold. This way, the amount of unnecessary wake-ups can be greatly reduced since bats that are more than 5 m away don’t trigger wake-ups anymore. The 5 m range of the wake-up receiver has been evaluated during field tests (see Section 4) in a mature forest environment by measuring the distance between which the nodes still have reception.

**Figure 4:**
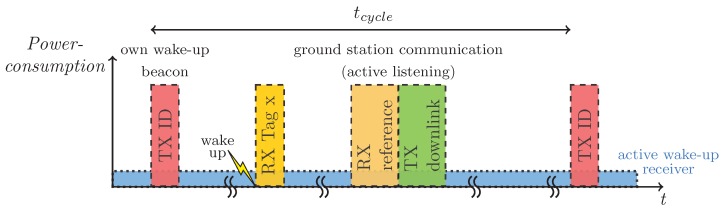
Wake-up receiver based communication scheme.

The mobile node periodically sends out its own On Off Keying (OOK) modulated wake-up beacons composed of the wake-up pattern and payload data like the own node ID as seen in red as “TX I” in Figure 4 with a transceiver power of 10 dB. Upon receiving such beacon from another node, the system wakes up (yellow lightning bolt), receives the remaining part of the beacon via the conventional receiver (orange) and triggers the data processing as described in Section 3.2. If a false wake up would be triggered, the following communication based on the conventional receiver would fail and no encounter is detected.

Since beacons are usually sent out every two seconds, the channel has a relatively low utilization even if multiple bats are present. Channel utilization is further optimized by automated reduction of the beaconing frequency in the roost which is the location with the highest probability of encounters. Thus, interference between nodes is not seen as a problem. If a packet collision should still occur, the beacon reception will fail and no encounter will be recorded. To increase the system robustness against package loss during meetings (due to beacon collision or other reasons) up to five beacons can get lost without interrupting the corresponding meeting. Depending on the current use case of the system these values (beaconing frequency depending on location as well as maximum beacon loss) can be adapted flexibly.

While, for proximity sensing, the short reception range of the wake-up receiver is an advantage, the usually high distance between the base station and the bats prevents the wake-up receiver to detect signals from the base station. Thus, to be able to receive the base station data, the node periodically turns on the conventional receiver to check for base stations in range. Other than the mobile nodes, the base station doesn’t have any strict energy constraints and the base stations are placed so that they can’t interfere with each other. This allows the base station to transmit during all available time slots. This way, the time the receiver is turned on doesn’t have to be synchronized with the base station, allowing the receiver to be active only a short period of time. Even though a conventional receiver is being used here, this way the energy consumption can be kept at a minimum. If a base station in range has been detected (marked dark orange in Figure 4) the *Base-Station Handler* described in Section 3.2 handles the communication.

#### 3.1.4. Power Management

Despite the limited weight and battery capacity, the sensor node should be powered for as long as possible. A lithium polymer battery with 25 mAh and a weight of 0.66 g has been chosen as power supply. Apart from a relatively high capacity in regards to its weight and size, the battery supports a peak current of around 25 mA. This is necessary to be able to power the radio to transmit/receive data. Compared to the used lithium polymer battery, coin prime cells are available in even higher capacities while still fitting the weight constraints. However, they only allow a low current draw. Since the active time of the node depends on received wake-up packages, the current consumption can not be predicted. Thus, a buffer solution that would smoothen the current peaks to suitable low levels can’t be used.

While the used NVRAM already has a much lower current consumption as comparable flash memory, it still draws the highest current on the mobile node. To further decrease the current consumption, the NVRAM can be cut off from the DC–DC converter by an internal control pin of the DC–DC converter. Thanks to the memory being non-volatile, it only has to be turned on during access and can kept turned off otherwise. A memory handling system which uses the SoC RAM whenever possible and only when necessary accesses the NVRAM is implemented in software to allow minimum on-time for the NVRAM.

**Figure 5:**
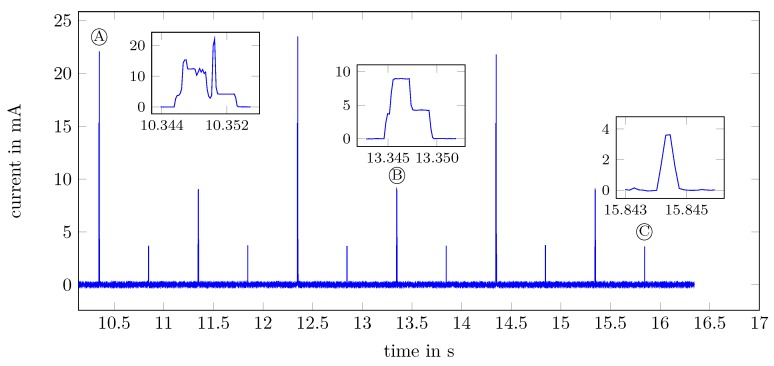
Measurement of the energy consumption of a BATS mobile node. The subplots next to the peaks marked Ⓐ, Ⓑ, Ⓒ show the detailed current consumption during these peaks.

Figure 5 shows a current measurement of the BATS mobile node. The measurement has been conducted in the lab without other mobile nodes or ground stations being present. The current was recorded with a Keysight N6705B power analyzer (Santa Rosa, CA, USA) equipped with a precision source.

The peaks marked Ⓐ occur every two seconds. They show the current consumption during calculating and transmitting the encounter data. This includes sending the OOK modulated wake up pattern as well as the Frequency Shift Keying (FSK) modulated transmission of the node ID. During all this, the system is active for 8 ms. Ⓑ corresponds to the mobile node turning on its receiver to listen if a base station is in range. If one is in range, the node transmits stored data to the station. Otherwise (like seen in the plot), if no base station is in range, the mobile node returns to a sleep mode after 5 ms. The current peak Ⓒ is caused by the Real Time Clock (RTC) periodically waking up the mobile node to increment the local time and perform various system tasks like checking for expired time-outs.

#### 3.1.5. Weight

To be able to measure the real behavior of bats and not influence the animal, it is crucial to keep the physical size and weight as low as possible. The overall weight constraint including the sensor node, battery and housing is below 2 g [20] and the size should be below 1 cm${}^{3}$ while keeping the height as flat as possible to reduce the impact on the bat.

To keep the PCB light enough, a 175 µm thin flex PCB substrate has been used. In addition, the size of the node is kept as small as possible and wherever feasible low weight components have been used. However, here it is important to find the right trade-off between weight and power consumption to allow a long enough runtime. The weight of the fully populated PCB is 0.510 g. The whole node including housing and battery has a total weight of around 1.3 g and thus undercuts the weight limit of 2 g by far. This enables the opportunity for a future integration of more functionalities in the sensor node hardware while still being usable on the investigated bat species. The low weight of the current system also enables expanding the scope of researched animals since now even lighter individuals can be tagged. By using a smaller 12 mAh battery the total sensor node weight can even be reduced to 1 g.

### 3.2. Software on Mobile Node and Its Static Analysis

#### 3.2.1. Software Overview

The hardware platform requires a specialized software, which orchestrate all peripherals in an energy efficient way. Therefore, minimizing overheads in terms of energy and computational effort is one of our most important goals. For an eased use and while also keeping overheads for context switches as low as possible, a custom fixed-priority alike scheduler is used. The biggest difference to a traditional fixed-priority scheduling is, that no preemption is supported. Due to a minimal context switch effort and no priority inversion problem, scheduling is as energy aware as possible.

**Figure 6:**
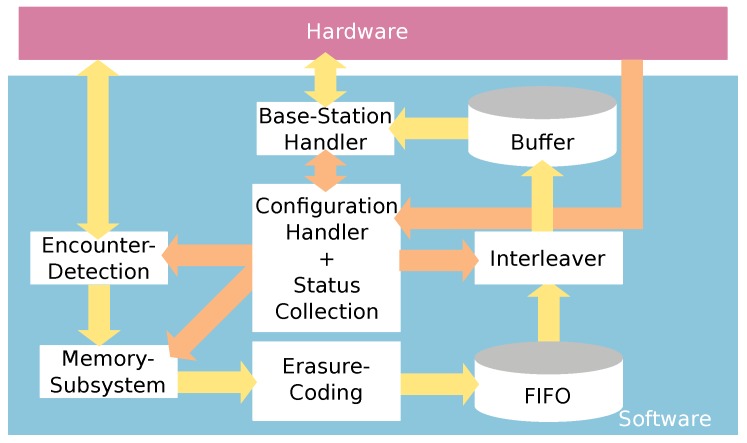
Overview of all submodules of the BATS application, running on the mobile node. Each submodule is decoupled by buffers to ensure an almost independent operation of all modules. The main data path is marked with yellow arrows and starts at the encounter detection and ends at the base-station handler.

The software can be divided into six modules, which are operating independently from each other due to buffering. In Figure 6, all modules are shown in an overview and is explained in the following.

The encounter detection derives data when an encounter took place and with whom. Furthermore, data on how long a meeting lasts and if the meeting is predominated with a bat that was resting are also collected. For better interpretations, a rough estimation on the distance in between both bats, the maximum RSSI value is logged. All nodes are sending out a so-called Mobile Node Beacon (MNB) periodically, which contains a wakeup-sequence and a unique identifier. In order to detect the presence of another bat in communication range, the wakeup-receiver is used. Once an MNB is received, the *Encounter-Detection* is invoked. This module looks up whether a MNB of this particular bat has been received recently and updates the values accordingly if this is the case. If no meeting is detected, the *Memory Subsystem* is used to allocate new memory to store the new meeting. Every second, the *Encounter-Detection* looks up, if five MNBs in a row were not received. If such kind of meeting is found, we consider the meeting as closed and push it into the memory in a first-in first-out memory, which then awaits transmission.

The *Memory Subsystem* is used to manage two different memory types, the internal Static Random Access Memory (SRAM) and the external NVRAM. If memory should be allocated, the internal SRAM is used to store data. This reduces overheads in terms of energy, as the NVRAM can be turned off as long as possible. However, if data should be stored on NVRAM, software transactions are used to prevent any corruptions of stored data due to transient failures like resets or power outages. This gives us the opportunity to store data among system resets.

If enough meetings were logged, the *Erasure-Code* module is invoked. The purpose of this module is to increase reliability of transmitted data by adding redundancy. In our case, we use a fixed code rate in which two meetings are encoded. Due to the erasure coding and our chosen code rate, two redundant packets are generated. The two redundant and two original packets are transmitted to a base station eventually. These four packets are later on called chunks, as a chunk plays a central role. Out of four packets in a chunk, all data can be reconstructed if at least two packets were received. Regardless of the pattern of received packets, using erasure-codes offers a better performance compared to simple duplication in terms of reliability.

The *Interleaver* is used to increase energy efficiency of the transmitted data. As the *Erasure-Code* adds redundancy, we interleave packets from different chunks to decrease overheads like starting the transmitter to a minimum. Thus, up to 35.14% of energy can be saved by an increased data rate while only negligible reliability is sacrificed. This module has been tested intensively theoretically and practically in multiple field tests, which is beyond the scope of this paper.

The last module inside the data path is the *Base-Station Handler* and decides whether a data transmission should be initiated and when. Depending on the current location of the bat or the node, the medium access is altered. This is because, if a bat is flying past a base-station, data transmission should be initiated with low latency to ensure a reliable communication. On the contrary, inside the roost, many nodes may send data to a base-station. As the bats are not moving inside the roost and due to the high communication efforts, it is beneficial to send data in a Time Divison Multiple Access (TDMA)-alike scheme. In order to detect the presence of a base-station, so-called Base Station Beacon (BSB)s are sent in a high frequency to the mobile node. Therefore, turning on the receiver can be done only for short times, which saves energy on the mobile node. The BSB contains data like the current configuration, the location and synchronization parameters which enables the mobile node to synchronize to the base-station.

The last module inside the application is the *Configuration Handler* and *Status Collection*. In order to ensure to alter a configuration only when no data is invalidated, the *Configuration Handler* keeps track of the whole application. With the reconfiguration, we are able to alter communication parameters like RSSI thresholds or timeouts. Furthermore, to monitor the whole system after deployment, we also collect statistics of the system like memory utilization or time of activity. This gives us the opportunity to alter the configuration, if unpredictable issues arise such as exhausted memory. If nodes are acting inappropriately, changing the configuration is the only way, as no reprogramming is possible after deployment.

#### 3.2.2. Static Resource Analysis

Energy management on the battery-operated mobile node is crucial in order to enable a sufficient lifetime for biological experiments. In order to estimate the lifetime, we developed analysis techniques to determine the worst-case energy consumption (WCEC) of operations (e.g., execution of specific tasks) [21,22]. These estimates are not only useful for the node’s lifetime estimation, but also to guarantee the completion of tasks, for example, when storing data to the available NVRAM.

**Figure 7:**
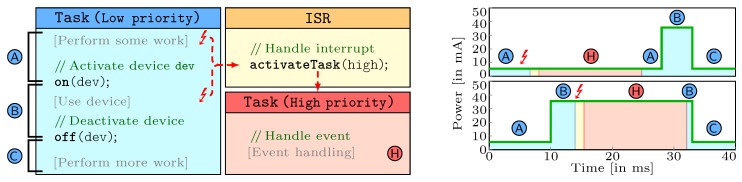
To determine upper bounds on the energy consumption of operations (i.e, execution of tasks) on the mobile node, temporarily activate devices and interferences of interrupts need to be considered.

The core challenges for the determination of WCEC estimates in the BATS scenario are twofold, that is, (1) dealing with temporarily activate devices and (2) considering interferences by concurrent activities in the analysis (i.e., possible interrupts, tasks with higher priority). Figure 7 exemplary outlines these challenges: a task of lower priority temporarily activates a device (e.g., the transceiver) and thereby leads to an increase of the node’s power consumption. In this scenario, an asynchronous interrupt can interfere the low task’s execution. Depending on whether the interrupt occurs within the low task’s duration of the activated device, the energy consumption significantly varies. The right part of Figure 7 illustrates these possible scenarios (i.e., high-power phase w/o interrupt). A safe WCEC analysis has to consider both scenarios and, in order to avoid unnecessarily pessimistic analysis results, has to precisely respect the phases where the device is switched off.

To solve these problems, we developed an analysis technique that captures phases of temporarily active devices and possible interferences in the context of the BATS projects [22]. In the first step of the analysis, we decompose the application code into blocks with a common set of active devices (see parts Ⓐ, Ⓑ, and Ⓒ in Figure 7). Using these decomposed blocks, we carry out an explicit path enumeration of all system-wide program paths that includes all interrupts and possible task switches. With knowledge of all possible paths, we formulate an integer linear program, whose solution eventually determines the upper on the energy consumption of the analyzed task.

### 3.3. Received Signal Strength-Based Localization

Recently, Received Signal Strength (RSS)-based Direction-of-Arrival (DOA) estimation techniques gained more attention in the research community. Several power-based approaches to direction finding have been published in literature. These may use multiple directional antennas [23,24], a single rotating antenna [25,26] or active reflectors [27]. An alternative opportunity, instead of mechanically moving the antenna, is the use of switched beam antennas as presented in Refs. [28,29]. Another approach is applying electronically steerable parasitic array radiator antennas, as presented lately in Ref. [30]. Recently, multi-mode antennas have been investigated for power-based DOA estimation [31]. In addition, a variant of the MUSIC algorithm for power measurements has been proposed in Ref. [32]. Theoretical limits in RSS-based direction finding have been discussed in Ref. [24].

For this paper, we consider RSS-based DOA estimation applying coupled dipole antennas [33]. Furthermore, it is assumed that localization takes place in the horizontal plane orthogonal to the two dipoles. Presuming a perfect linear dipole array, the radiation of the dipoles in the horizontal plane (i.e., $\theta =90^{{}^{\circ}}$) is constant over all impinging signal angles in azimuth ϕ∈[0,2π]. Hence, the radiation pattern for *N* dipoles is given by the array factor [34] $\mathrm{AF}=\sum_{i=0}^{N-1}c_{i}\cdot \exp\left(-j\cdot 2\pi \sin\left(\theta \right)\cdot d_{i}\cos\left(\phi \right)\right),$ where $d_{i}$ are the corresponding distances of the dipole elements, λ is the wavelength, and $c_{i}$ is the coupling factor. Considering only two dipoles at distances $d_{0}=0$ and $d_{1}=d$ and $\theta =90^{{}^{\circ}}$ the array factor reduces to $\mathrm{AF}=c_{0}+c_{1}\cdot \exp\left(-j\cdot 2\pi \cdot d\cos\left(\phi \right)\right).$

For the considered antenna array, the dipoles are coupled in phase and out of phase, respectively. Hence, the radiation patterns are given by Ref. [24] $g_{0}\left(\phi \right)=1+\exp\left(-j2\pi d\cdot \cos\left(\phi \right)\right),\;\mathrm{and}\;g_{1}\left(\phi \right)=1-\exp\left(-j2\pi d\cdot \cos\left(\phi \right)\right).$ We define the radiation power patterns $G_{a}\left(\phi \right)$ (in dB) by $G_{a}\left(\phi \right)=10\mathrm{lg}{\left|g_{a}\left(\phi \right)\right|}^{2}.$ The gain difference function of the described antenna array is expressed by

(1) $$\begin{array}{c} \Delta G\left(\phi \right)=G_{1}\left(\phi \right)-G_{0}\left(\phi \right). \end{array}$$

**Figure 8:**
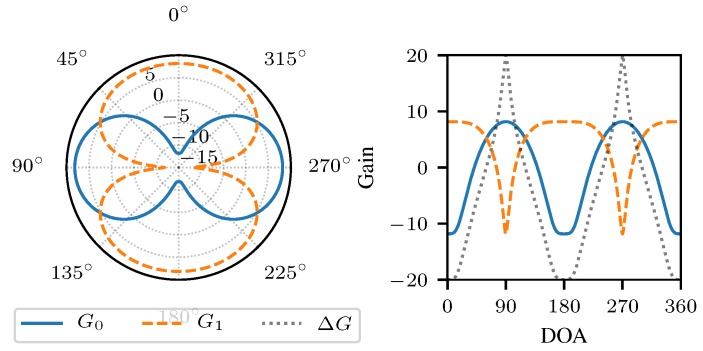
Radiation power patterns for perfect dipole antennas in horizontal plane for in phase and out of phase coupling.

The radiation power patterns for the antenna array at hand and the gain difference function are depicted in Figure 8.

The RSS at a receiver *a* for a transmitted signal with power $P_{\mathrm{TX}}$ can be computed as follows: $P_{\mathrm{RX},a}=P_{\mathrm{TX}}-L+G_{\mathrm{TX}}+G_{a}\left(\phi \right),$ with *L* denoting the bulk path loss. $G_{\mathrm{TX}}$ and $G_{a}\left(\phi \right)$ are transmit and receive antenna gain, respectively. When considering a single signal source, i.e., no multipath propagation, the received signal strength difference is given by

(2) $$\begin{array}{c} \Delta P_{\mathrm{RX}}=\Delta G\left(\phi \right)+w, \end{array}$$

due to the fact that both channels are stimulated by the same transmit power and exhibit equal path loss. Thus, the gain difference function does not depend on transmit power and path loss. Hence, it may be estimated without prior knowledge of the the path loss exponent and the power emitted by the transmitter. This fact is, in contrast to range-based localization based on RSS, a major benefit of RSS-based DOA estimation. The above consideration holds for the absence of multipath propagation. In case of multiple Multipath Component (MPCs), the observed difference in signal strength is not linked to the DOA of the Line-of-Sight (LOS) component.

**Figure 9:**
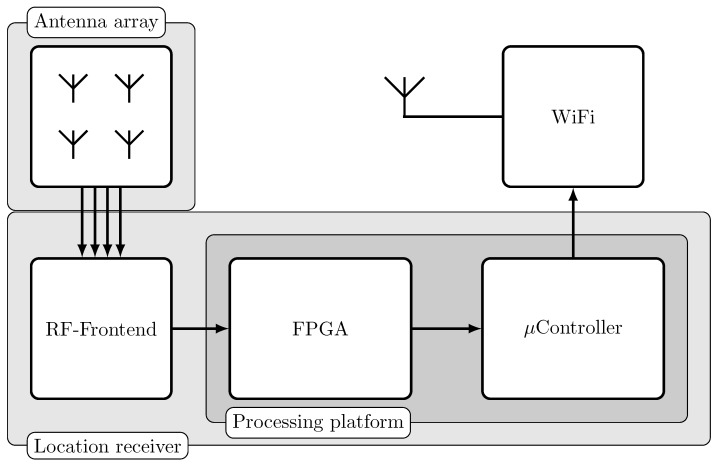
Block diagram of sensor node.

**Figure 10:**
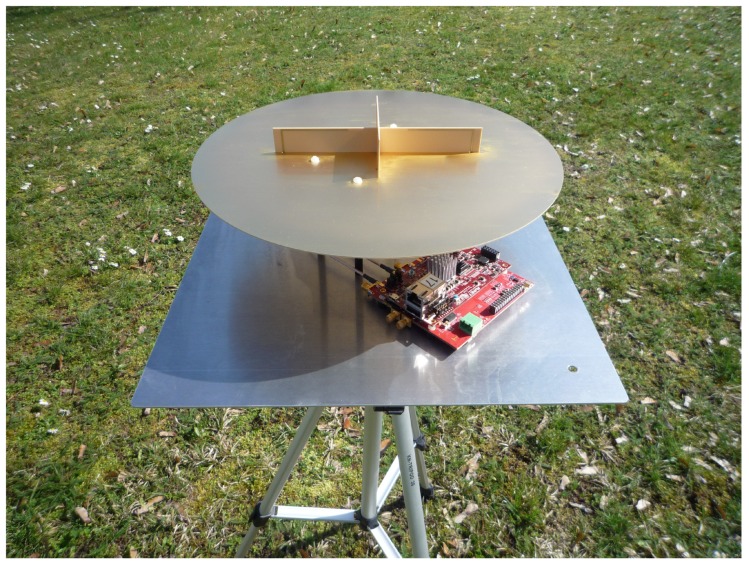
Localization receiver.

For an improvement of the localization accuracy, every localization sensor node utilizes two frequencies. The developed antenna for our application is presented in [33] and has two orthogonal antenna pattern for the frequencies 868 MHz and 2.4 GHz. Due to the large frequency distance, the fading of the two frequencies can be assumed as uncorrelated. In Figure 9, a block diagram of a localization sensor node is shown. The antenna array is connected to a RF-frontend which receives at 868 MHz and 2.4 GHz simultaneously with two channels. In the Field Programmable Gate Array (FPGA) of the processing platform, the signal detection is performed by correlation to the preamble and sync word of the mobile node signal like presented in [33]. The detected signal is processed by the microcontroller where a frequency estimation and correction is performed to decode the bat ID and data. In Figure 10, a picture of a localization node is shown. During operation, the antenna and receiver are covered with a housing.

#### 3.3.1. Optimal Design of RSS-Based DOA Sensors

As the classical performance measures, such as the Cramer–Rao Lower Bound, are not capable of considering ambiguities in DOA estimation, power-based DOA estimation sensors are reviewed from an information-theoretic view in this section. The basic idea is find a antenna geometry that maximizes the information gained from an RSS measurements [35]. Recently, there has been a revival of information theory in many fields. Information-theoretic measures have been utilized, just to a name a few of them, to optimize Multiple Input Multiple Output (MIMO) radar waveforms [36], to quantify the loss in sub-Nyquist sampling [37,38], and to compute fundamental limits in compressed sensing [39] and bounds for kernel-based time delay estimation [40]. Utilizing the framework of information theory, this can be achieved maximizing the mutual information. Maximizing the mutual information, i.e., the total information gained from a sensor measurement, is by far more generic than local precision measures, such as the Cramer–Rao Lower Bound (CRLB). Thus, in contrast to the CRLB, information-theoretic measures are applicable in case of multi-modal or non-Gaussian probability densities [35].

Compared to the most common approach directly inferring DOA from phase differences, such as uniform linear antenna arrays, in RSS-based DOA estimation, coupling between antenna elements is used to realize angle dependent gain patterns. This can be thought of as static beam forming. The benefit of this approach is that DOA of the impinging signal results in an RSS change at the receiver. Hence, DOA estimation is feasible with non-coherent receive channels. From an economical point of view, this is very beneficial since non-coherent receiver may be manufactured at a low cost compared to phase-coherent receivers. A realization of such a low-cost tracking system has been presented in Ref. [33].

The major challenge is the design of the antenna radiation patterns of such a localization system. The shape of the antenna pattern is defined by the geometrical arrangement of the antenna elements, the gain pattern of the elements and the combination of the signals. In the sequel, the mutual information of a DOA measurement considering the BATS antenna is computed. The following setup is considered. Localization takes place in the horizontal plane. The antenna array consists of two dipoles that are orthogonal to the plane, i.e, $\vartheta =90^{{}^{\circ}}$. The two dipoles are coupled in phase and out of phase for the two antenna ports as described in the section above and the gain pattern as described in Section 3.3. Previously, a distance between the dipoles of d=λ2 was considered. In this section, the distance *d* is the design parameter of the antenna array to be optimized. The criterion to be maximized is the mutual information. In other words, the distance with the maximum total information gain is sought.

In a nutshell, the following procedure needs to carried out in order derive the optimal array geometry for power-based DOA estimation:

1. Compute radiation patterns for distance *d*,
2. Derive measurement function, i.e., the gain difference function ΔG,
3. Compute posterior Probability Density Function (PDF) and joint PDF,
4. Compute entropy.

**Figure 11:**
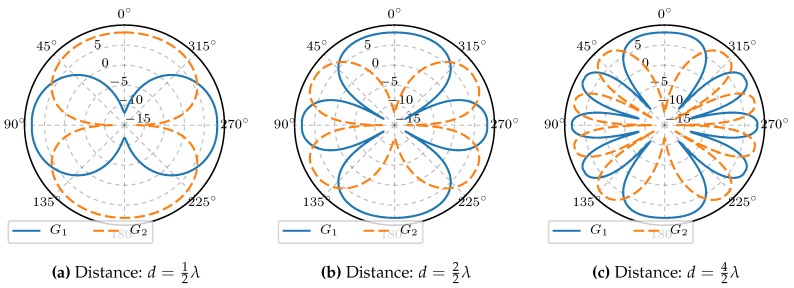
Radiation power patterns for antenna arrays with different dipole distances.

Radiation power patterns for different distances between the two dipoles are shown in Figure 11. Obviously, the in phase and anti-phase feeding results in orthogonal radiation patterns. Furthermore, it is easy to recognize that an increasing number of lobes results in an increasing gradient of the gain difference function ΔG. That in turn leads to a decrease in the estimation variance in the presence of measurement noise. However, on the other hand, ambiguities arise.

We now consider information-theoretic measures to optimize the antenna array described above. In RSS-based DOA estimation the measurement likelihood is given by $p\left(\Delta P_{\mathrm{RX}}|\phi \right)∼N\left(\Delta G\left(\phi \right),\sigma_{\Delta P_{\mathrm{RX}}}^{2}\right).$ It is assumed that there is no prior information on the signal direction available. Hence, a non-informative prior is chosen p(ϕ)∼U(−π,π). With the prior the entropy before the RSS difference measurements is given by h(ϕ)=−∫p(ϕ)log(p(ϕ))∂ϕ. The conditional entropy is computed as follows:

(3) $$\begin{array}{cc} h(\phi |\Delta P_{\mathrm{RX}}) & =-\;\int \int \;p\left(\phi ,\Delta P_{\mathrm{RX}}\right)\log\left(p\left(\phi ,\Delta P_{\mathrm{RX}}\right)\right)\partial \phi \partial \Delta P_{\mathrm{RX}}, \end{array}$$

with the posterior PDF given by $p\left(\phi |\Delta P_{\mathrm{RX}}\right)=p\left(\Delta P_{\mathrm{RX}}|\phi \right)p\left(\phi \right)/p(\Delta P_{\mathrm{RX}})$ and joint PDF expressed by $p\left(\phi ,\Delta P_{\mathrm{RX}}\right)=p\left(\Delta P_{\mathrm{RX}}|\phi \right)p\left(\phi \right)$ Finally, the mutual information is given by

(4) $$\begin{array}{cc} I(\phi ;\Delta P_{\mathrm{RX}}) & =h\left(\phi \right)-h\left(\phi |\Delta P_{\mathrm{RX}}\right). \end{array}$$

The mutual information quantifies the total information gained from an RSS difference measurement.

**Figure 12:**
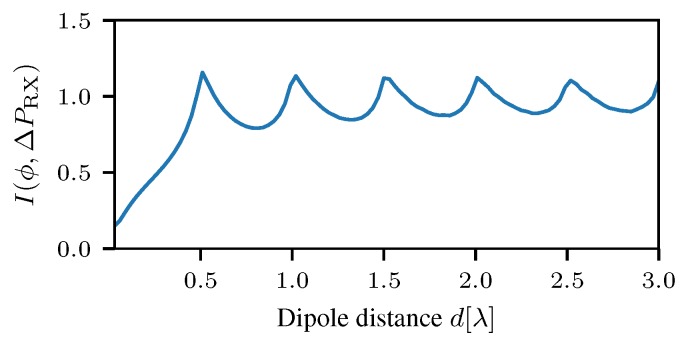
Mutual information of Received Signal Strength (RSS)-based Direction Of Arrival (DOA) estimation for two orthogonal patterns at different dipole distances.

In Figure 12, Equation (4) is evaluated for different distances between the two dipoles. As with the phase-based direction finding, the mutual information increases for distances increasing from 0 to λ2. At a distance of λ2, the mutual information has a maximum. Beyond λ2, the mutual information decreases until it increases again towards a distance of λ. It can be seen that the mutual information has local maxima at distances d=n·λ2. Apparently, all local maxima have the same height. Hence, the optimal dipole distance is dopt=n·λ2. In conclusion, it is possible to trade ambiguities for local precision or vice versa without changing the total information gained from RSS difference measurements. In other words, all DOA sensors with d=n·λ2 provide exactly the same information.

#### 3.3.2. Multipath-Robust Localization

In this section, a probabilistic multipath mitigation method is presented that makes use of statistical prior channel knowledge in order to compensate multipath effects [41]. The presented mitigation technique allows for mean-free DOA estimates on average if prior knowledge of the channel parameter Angular Spread (AS) is available. This is achieved by computing multipath adaptive power patterns for the utilized antenna arrays incorporating the AS. As mentioned in Section 3.3, bulk path loss and shadowing have no influence on the RSS difference at the receive antenna. However, multipath propagation affects the RSS difference significantly. Hence, the measured RSS difference is impaired that degrades the DOA estimation.

In general, the impulse response of a wireless channel can be described by a tapped delay line [42] of *L* MPCs given by $h\left(t\right)=\sum_{l=1}^{L}a_{l}\cdot \delta \left(t-\tau_{l}\right),$ where $a_{l}$ is the complex coefficient of the *l*-th multipath component and $\tau_{l}$ its respective delay. Considering the presented antenna array, the RSS difference of a received signal affected by multipath propagation can be calculated by superposition of the MPCs

(5) $$\begin{array}{c} \Delta P_{\mathrm{RX}}=10\mathrm{lg}{\left|\sum_{l=1}^{L}g_{1}\left(\phi_{l}\right)\cdot a_{l}\right|}^{2}-10\mathrm{lg}{\left|\sum_{l=1}^{L}g_{2}\left(\phi_{l}\right)\cdot a_{l}\right|}^{2}, \end{array}$$

where $g_{r}\left(\phi_{l}\right)$ is the complex gain coefficient of antenna *r* at the arrival angle $\phi_{l}$. With Equation (5), the effective gain of the receive antenna for a particular realization of the multipath channel can be computed. Obviously, the angular spread significantly impairs the measured RSS at the two antennas and thus causing a bias in DOA estimation. In the presence of multipath propagation, Equation (2) does not hold true. In multipath scenarios, there is no distinct relation between gain difference and RSS difference in general, and hence

(6) $$\Delta P_{RX}\neq \Delta G\left(\phi \right)+w.$$

The basic idea of mitigation technique is to derive a modified measurement function, i.e., and adaptive gain difference function $\Delta G_{\mathrm{MPC}}\left(\phi \right)$, that allows for compensation of the multipath induced bias on the DOA estimates. For the multipath adaptive gain difference function, $\Delta G_{\mathrm{MPC}}\left(\phi \right),$ the following equation has to hold true:

(7) $$\Delta P_{RX}=\Delta G_{\mathrm{MPC}}\left(\phi \right)+w.$$

If a gain difference function $\Delta G_{\mathrm{MPC}}\left(\phi \right)$ can be found that fulfills Equation (7), multipath impairments can be mitigated in a probabilistic manner.

**Figure 13:**
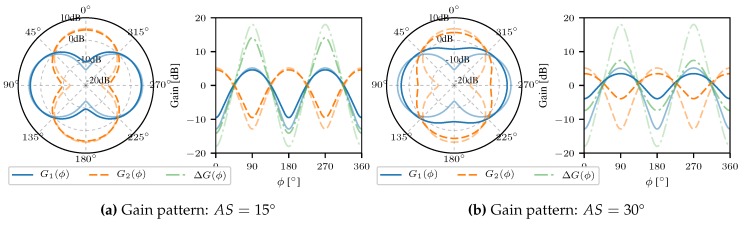
Multipath adaptive models describing the expected RSS difference for different values of angular spread. Dotted lines denote the original pattern.

This RSS-based DOA is realized by incorporating the angular spread in the description of the antenna patterns. Such a multipath adaptive measurement function realizes a DOA estimation that has a zero-mean error on average. Therefore, the circular gain patterns are convolved with a scaled normal distribution with a standard deviation of $\sigma_{\phi_{\mathrm{AS}}}$. Hence, the multipath adaptive measurement model is derived as follows ${\left|g_{\mathrm{MPC}}\left(\phi \right)\right|}^{2}={\left|g(\phi )\right|}^{2}*p\left(\phi \right),$ where p(ϕ) is the PDF of the distribution of the angular spread given by $p\left(\phi \right)∼N\left(0,\sigma_{\phi_{\mathrm{AS}}}^{2}\right),$ and (∗) denotes a circular convolution. Note that the gain function is in non-logarithmic scale here. The resulting gain patterns are depicted in Figure 13 for different values of the angular spread. For smaller spreads the modified measurement function does not differ much from the gain difference function of the original antenna patters. Larger spreads result in flattened effective gain functions. It can be easily seen that the dynamic range of the effective gain difference function is significantly reduced due to a larger AS, i.e, the presence of multipath. Under the assumption that the channel parameter AS is known, it has been shown that probabilistic multipath mitigation allows for zero-mean DOA estimates.

**Figure 14:**
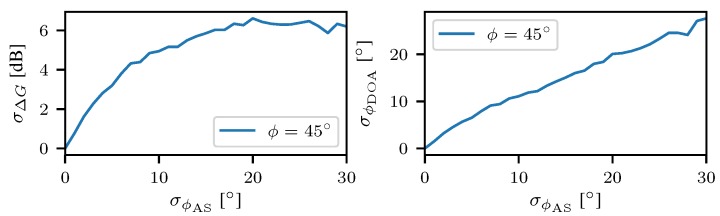
Standard deviation of RSS difference due to multipath propagation (**left**). Standard deviation of the inferred DOA estimate (**right**).

The presented approach allows for zero-mean compensation of effects from multipath propagation [41]. However, that mitigation does not come for free and leads to an inherently increased variance in RSS difference measurements. The dependence of RSS difference standard deviation has been determined by Monte Carlo simulations and is depicted in Figure 14 for a LOS angle of $45^{{}^{\circ}}$. Observing the results presented in Figure 14, the standard deviation for the RSS difference is saturated at mean spreads larger than $20^{{}^{\circ}}$ and resides constant at a values of ∼7 dB. One might conclude that the variance of the RSS inferred DOA also does not increase with larger angular spreads. However, that is a false conclusion. The multipath adaptive gain difference function flattens with increasing angular spread (cf. Figure 13) which results in a decreasing gradient of the function $\Delta G_{\mathrm{MPC}}\left(\phi \right)$. Thus, the variance of the DOA estimate increases according to the linear transformation of the variance from measurement domain to parameter domain $\sigma_{\phi_{\mathrm{DOA}}}\left(\phi_{0}\right)\approx {\Delta G}^{-1}\left(\phi_{0}\right)\cdot \sigma_{\Delta P}\left(\phi_{0}\right).$ Hence, with increasing mean angular spread even for a constant variance in RSS difference the variance in DOA estimates increases. The proposed multipath mitigation technique enables for unbiased DOA estimation in multipath scenarios with known mean angular spread. However, the mitigation comes at an expense of an increased variance in DOA estimates.

### 3.4. Long Range Telemetry

As already addressed in Section 3, the ground network enables the download of encounter data, so-called meetings and provides a precise localization of bats in the close-up range. However, the trackable region is limited to the area covered by the base station network. The usage of additional stations is costly, both in acquisition and operation effort. With the bats flying at high speed, they are likely to leave this region within a short period of time, especially when hunting. While no high-resolution tracking can be provided outside the ground network, the bats shall still be observable by the system even in distances of several kilometers to enable a long-term monitoring. To tackle this challenge, a dedicated system of distributed telemetry base stations was established, forming a Low Power Wide Area Network (LPWAN). Theoretical analysis [43] as well as field results [44] demonstrate, that low rate sensor data like bat identification, air pressure, 3D-acceleration or other sensor data can successfully be received kilometers beyond the ground localization network (see Section 4). In the following, we briefly illustrate the system components and algorithms implementing the long range telemetry transmissions.

#### 3.4.1. Long Range Telemetry Transmission Scheme

For low power long range data transmission there are already systems like LoRa or Sigfox available. However, LoRa as well as other systems require sending dedicated packages for long range communication. The transmitter nodes are limited both in size and weight since the bat is not capable to carry larger batteries. Therefore, incorporating a long range telemetry scheme is imposing rather contradictory requirements on the system since no additional hardware or energy source should be added. However, an additional long range transmission shall be implemented. Therefor the BATS project makes use of already transmitted packages rather than adding an additional ones. The encounter detection in the BATS project (see Section 3.1.3) is performed utilizing an OOK modulation scheme with limited range. When this, so-called wake-up signal (cf. Figure 3, component *WuRX*), is received by another bat near by, it triggers its processor to return from deep sleep mode for an exchange of bat IDs and metadata, noted as encounter or meeting.

**Figure 15:**
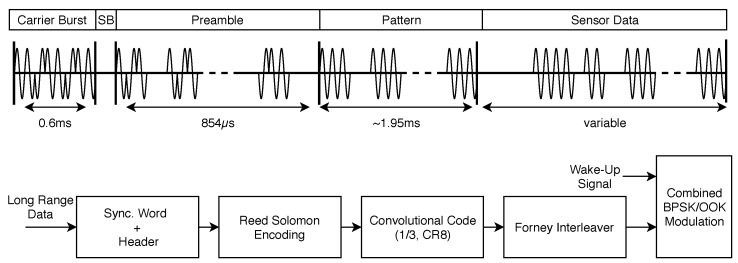
Combined modulation of long range telemetry data utilizing the wake-up burst showing the corresponding waveform and encoding scheme.

In order to embed the telemetry transmission into the existing system, we perform a combined modulation of OOK-modulated wake-up bits together with a Binary Phase Shift Keying (BPSK) modulation for encoding the telemetry data using two BPSK bits per OOK bit. This way additional packages are omitted and the energy efficiency of the developed long range scheme is far more efficient than state of the art systems that require their own packages. Figure 15 depicts the encoding scheme and waveform for the telemetry transmission proposed, to cope with the harsh restrictions on energy and weight.

As can be seen in the upper part of the Figure, the OOK-modulated signal is alternated in its phase at distinct sample instances. This is done during the carrier burst and preamble of the wake-up pattern (cf. Figure 15), as these sequences are common to all bat nodes, fixed in length and structure and also the on-times are known. The pulses are Manchester-coded to eliminate any dependency between the number of on-times and the data transmitted within the wake-up signal. This allows for an efficient exploitation of this OOK scheme for implementing the long range telemetry transmission without supplementary hardware or an additional expenditure of energy, as the phase is not used by the wake-up itself.

For a successful decoding even in large distances, a sophisticated encoding must be applied. Following the lower part of Figure 15, the long range payload data is extended by a synchronization word and header information carrying the bat ID among other information. Error detection is performed by means of a shortened Reed-Solomon Code RS(253,255) in combination with a convolutional code (0255,0331,0367) exhibiting a code rate of $\frac{1}{3}$ and a constraint length of 8. A Forney interleaver with 24 branches distributes one payload byte over 24 wake-up bursts, resulting in a time interleaved transmission of each long range telemetry packet of almost one minute. This approach adheres to the idea of the so-called Telegram Splitting concept as presented in [45,46]. Thereby, information is spread within the time (and frequency) plane in a burst-like fashion. As the bat nodes operate in the unlicensed Short Range Devices (SRD) band around 868 MHz, this technique is eligible to mitigate the influence of the channel and other interferers, as experienced in earlier measurements [47], thus assuring an ultra-robust transmission in combination with the Forward Error Correction (FEC) algorithms presented. Given this encoding, we obtain a nominal payload data rate of 11.363 kbit/s for the telemetry modulation scheme. Accounting for the duty cycle (wake-up burst length of about 3 ms) along with the error coding overhead, one results in one absolute payload byte per burst or a rate of about four payload bits per second (under the presumed configuration of a 2 s burst interval). Thus, a low rate and robust long range telemetry transmission is implemented, capable of transferring periodically gathered sensor data without the need for additional energy or any system changes, except for software.

#### 3.4.2. Telemetry Base Station Architecture

**Figure 16:**
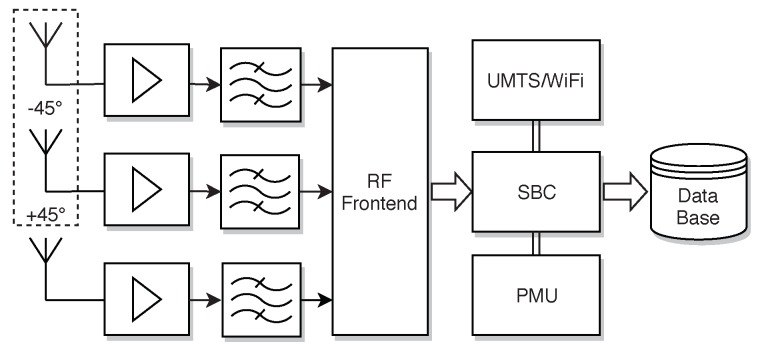
Simplified block diagram of the long range telemetry base station architecture.

For an optimal system performance, the receiving network also has to be properly designed. In Ref. [43], we presented theoretical analysis of the achievable transmission range related to rate and the environment scenario modeling the radio channel. Depending on the height of the transmitting bat nodes and the receiving base stations, one has to overcome path losses of more than 150 dB in distances of 5 km, while supporting data rates of just a few bit/s for a balanced relation of energy expenditure per telemetry bit. This finding is in compliance with our system layout as described in Section 3.4.1. To alleviate the influence of shadowing obstacles like trees, the base stations, forming the long range telemetry reception network, are located at exposed sites around the habitat of bats. Measurement sites on roof tops or towers assure an almost line of sight connection to the bats when flying. Figure 16 shows a simplified signal processing chain of a telemetry base station.

The station is equipped with three antennas in total, where one is a directional antenna internally consisting of two cross-like antenna arrays rotated against each other (cf. $\pm 45^{{}^{\circ}}$). These antennas exhibit with a high average gain of 14dBi at a half-power beam width of $66^{{}^{\circ}}$ to counteract the high losses to be expected. A third omni-directional antenna with 0dBi assures a gap-less spatial reception coverage. This MIMO like architecture enables means of stream combining and beam forming to mask interferers and further improve the decoding rate. Each reception stream is fed through a Low Noise Amplifier (LNA) and a half-band filter cascade, before being digitized by a self-developed radio frontend. A C++ driven Software Defined Radio (SDR) framework [48], hosted by a single board computer (SBC), performs signal processing before compressing and stores the In-Phase and Quadrature (IQ) data to a local persistent storage. A Universal Mobile Telecommunication System (UMTS) modem provides means for remote control, while the Power Management Unit (PMU) controls the discharge and recharge process via solar panels of the station’s batteries, allowing for an autonomous and self-sustaining operation.

**Figure 17:**
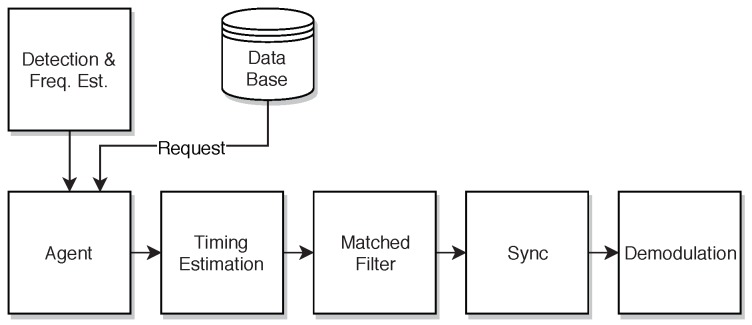
Offline demodulation process of long range telemetry data.

Figure 17 illustrates the demodulation process of the gathered data. With three data streams, each exhibiting a sampling rate of 2 MHz and a resolution of 12 bit for in-phase and quadrature component, respectively, the resulting transfer rates for streaming the recorded data to a remote storage via the internet would be costly and would constitute a bottleneck for the digital signal manipulation. The signal processing chain has to cope with these high rates to avoid data loss by buffer overflows. Therefore, the demodulation is performed offline. With the vast amount of raw IQ data, a direct search for telemetry signal bursts would be both time and computationally intensive. To alleviate this problem, we implemented a so-called *Agent* (cf. Figure 17). This software module is instantiated for each bat signal, detected by a preceding detection algorithm. We exploit the periodicity of bursts. As described in Section 3.4.1, the long range data is structured in packets that are distributed over several wake-up bursts and encoded within phase changes. Therefore, only after the demodulation of several subsequent bursts, one complete telemetry packet is retained again (compare *Telegram Splitting* technique). Thus, once a signal has been discovered, the *Agent* can make concise requests to a database, rather than loading and traversing complete streams. The database in turn provides metadata, such as a precise Coordinated Universal Time (UTC) timestamp, which eases the access, following the periodic burst sequences in time intervals of several seconds. Subsequently, a multi-stage timing and frequency estimation are carried out, followed by matched filtering, synchronization and the demodulation process.

This section briefly illustrated the long range telemetry functionality. The new transmission scheme was successfully integrated into the existing system without the need for additional hardware on the sensor nodes or an increased energy consumption. We presented both the introduced telemetry base stations as well as the software components running the transmitter and receiver side. First measurement results are given in Section 4, to prove the long range functionality in a practical setup.

### 3.5. Data Backend

Meeting data must be cleansed before analysis. There is an inconsistency between the recorded meeting data and domain knowledge. On a semantic level, meetings between tracked objects are reflexive, i.e., if object 5 has met object 7, object 7 also must have met object 5. However, often the counterparts are missing in the recorded data.

Another issue is a classification issue. If other means are not available, the position can be deduced from the number of simultaneous meetings. Trajectories are measured in an a priori determined area of interest and are thus not usable to determine positions outside of this area. We often used the ID of the base station which received the meeting data to deduce locations. However, in some scenarios, it is not feasible to install a base station at certain locations because they are not known a priori, or they are simply hard to reach. In the case of bats, one very interesting location is the roost. We expect, more generally, that locations of assemblies are interesting in most scenarios. Even if we cannot determine the exact location of an assembly, it is still valuable to know when a tacked object participated in an assembly. To do this, we use the number of simultaneously met objects to decide whether an object was at an assembly or not.

**Figure 18:**
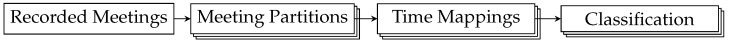
Overview of the Data Backend Processing Steps.

Figure 18 illustrates these computational steps. All steps can be performed in parallel for each ID. This is indicated by shadows.

#### Implementation

To solve the inconsistency issue, either meetings lacking their reflexive counterpart must be removed or the missing counterparts must be added. As the meeting detection does not create false positives, adding missing values is the correct approach.

However, instead of physically adding missing counterparts to the data set, we make use of the fact that the data must be partitioned for later classification anyway. During partition creation, we use all reported meetings involving the current ID of interest regardless of the reporters’ IDs, and thereby erase the information of origin. This results in one consistent table of possibly overlapping meetings for each ID.

**Figure 19:**
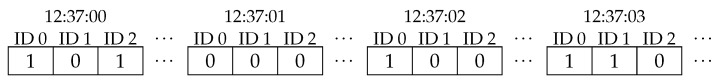
Memory layout of the mapping from time to the set of encountered Ids.

The next step is to create a mapping from time to the currently encountered ids for each object. This implicitly removes the redundancy created by overlapping meetings. We represent this mapping by creating an appropriately sized array of bitsets. Each bitset represents a second, and each bit in the bitset corresponds to an ID of a potential meeting partner (cf. Figure 19).

**Figure 20:**
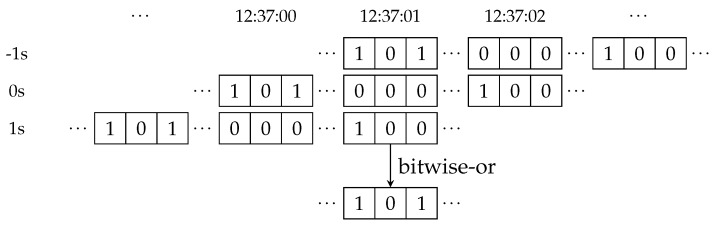
Union operations for a two-second window.

As the data set contains a lot of noise, the results are smoothed by considering windows of user-specified size centered around each second. The results are stored in a second array whose content is the union of all bitsets in the corresponding window. This is implemented efficiently by virtually shifting the input array by offsets and using the bitwise-or operation to implement the set-union. Figure 20 illustrates this operation by continuing the example from Figure 19 for a two-second window around 12:37:01. Finally, the assembly classification is done by comparing the number of set bits to a specified threshold.

### 3.6. Diversity Combining for Improved Communication

Whenever a bat equipped with a sensor tag visits the hunting ground, the stored contact information needs to be transmitted down to the deployed ground network. As the communication takes place in a forest environment, the signal is not only affected by Free Space Path Loss (FSPL) and multi-path fading, but, additionally, by shadowing from trees that lie in between. Furthermore, due to the fast movement of bats and low transmit power of the sensor tag, there is a need of techniques that offer increased communication reliability. Since there are already multiple base stations available in the ground network for localization purposes, there is a high probability that the signal transmitted by the bat sensor tags will be received by multiple of these base stations. Therefore, we propose to use these base stations in the ground network as a distributed antenna array to apply receive diversity combining for an improved reception.

Using spatially separated antennas on the receiver side to perform diversity combining is one of the most popular and cheap techniques to combat fading. The most commonly used diversity combining techniques include Maximum Ratio Combining (MRC), Equal Gain Combining (EGC), and Selection Diversity (SD) [49]. On the one hand, MRC and EGC provide the highest diversity gain but that comes with an expense of increased processing demands. For example, to perform constructive combining, all branches are weighted (unity for EGC and relative to their received Signal-to-Noise Ratio (SNR) for MRC), aligned, and co-phased before addition. While, on the other hand, SD selects a branch with the highest Signal-to-Noise Ratio (SNR) and, hence, does not require a complex algorithm, but the diversity gain achieved is also minimal compared to the others.

Such a distributed antenna system also helps to make the system becomes more robust against not only multi-path fading but also shadowing [50]. This concept is similar to macro-diversity used in cellular networks in which multiple base stations connected to each other via optical fiber cooperatively decode the same signal to increase the RSS [51]. In our system, nodes in the ground network are connected to each other through wireless connections, hence, it imposes a limit on the maximum data rate that is achievable to exchange the information between nodes to perform diversity combining at a single point.

One option to successfully forward data from all base nodes to a sink node is to process the received data at nodes locally and forward soft-bits only [52]. Since soft-bits contain only one float value for every single bit, the information that needs to be forwarded reduces dramatically, hence, the network is not overloaded. The sink node combines soft-bits instead of signal samples, which certainly increases the RSS. However, the diversity gain achieved is not the highest because the system loses signal properties while converting the signal into soft bits [53]. As an alternative, we propose the concept of selective signal sample forwarding [54]. In the proposed approach, all base nodes detect the received signal copy locally by correlating the incoming samples with the known preamble. In the case of detection, channel parameters such as phase information is estimated also through the preamble and compensated for constructive combining. The local nodes then slice the signal starting from preamble equivalent to the known packet length and forward only these relevant I/Q samples to the sink. As multiple bats can transmit with a maximum rate of 100 Hz, a packet size of less than 1 ms reduces the data rate required in the ground network by more than 10 times and, thus, resulting it in the range of few Mbit/s. Finally, the sink node receives all locally detected signal copies and applies diversity combining on the received I/Q samples. The performance achieved with the selective signal sample forwarding is the same as achieved with the conventional diversity combining and degrades only if the local base nodes do not detect the signal successfully [54].

**Figure 21:**
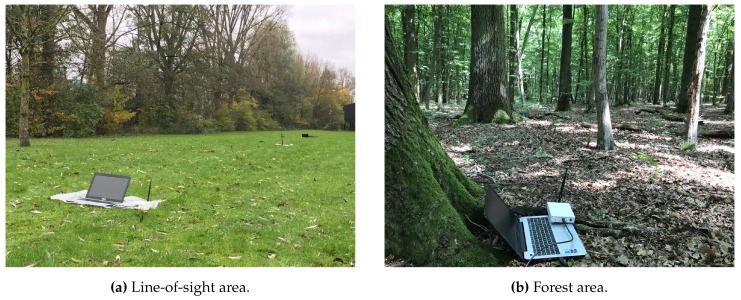
Types of areas to conduct diversity combining experiments.

To analyze the performance of our proposed approach, we implemented the BATS transmitter and base nodes receiver in GNU Radio, an open source signal processing tool to implement the software part of a radio. We validated our implementation by conducting an extensive set of simulations and over-the-air lab measurements. The implementation and validation details are explained in [55]. As a step further, to test our model in realistic environments, we performed a series of outdoor experiments in LOS and forest areas as shown in Figure 21. To conduct experiments, we use two Ettus B210 Universal Software Radio Peripheral (USRPs) (Santa Clara, CA, USA) as receivers and one as a transmitter all connected to laptop computers. We placed both receivers 30 m apart and moved the transmitter at a human walking speed, i.e., 4 km/h to 5 km/h, between the receivers in such a way that its distance from both receivers always remains equal during an experiment in both areas. The maximum transmit power of B210 USRP is in the range of 10 dBm; however, since the USRPs are not perfectly calibrated, we fixed the transmit power by adjusting the gain in a way that the average Packet Delivery Ratio (PDR) at a single receiver stays better than 50%. We recorded the data of each receiver to apply various diversity techniques with exactly the same channel conditions and processed it offline.

**Figure 22:**
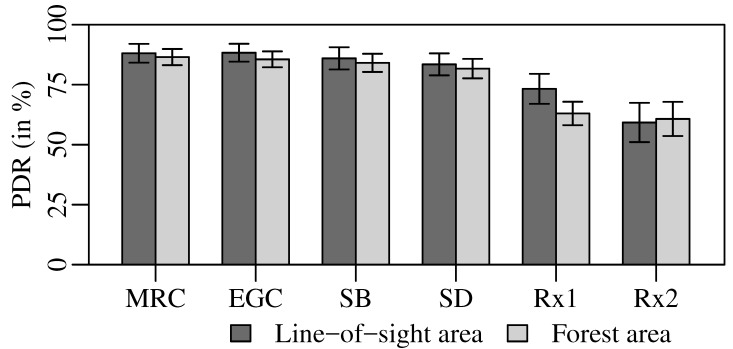
Packet delivery ratio achieved with different diversity combining techniques in realistic environments.

Figure 22 shows the PDR achieved at each individual receiver, i.e., R × 1 and R × 2, as well as results for the different diversity techniques. The error bars depict the 95% confidence intervals and are obtained by repeating the experiments 30 times. As SNR in the resultant signal in MRC is the linear combination of SNRs of individual signal copies, it provides the best performance, i.e., achieves a PDR of 88% and 86.5% in LOS and forest area, respectively. EGC performs only marginally worse than MRC despite the fact that EGC involves relatively less processing to calculate the gain values. This happens due to the fact that only two receivers or branches are involved with roughly similar SNR. The difference between MRC and EGC is more prominent if higher numbers of branches contribute for diversity combining. Successful Branch (SB) represents the performance of a system in which a reception is considered successful if any of the base nodes decode the signal correctly without any combining. SB performs inferior to MRC as well as EGC and achieves a PDR of 86% and 84% in LOS and forest area, respectively. Nevertheless, the performance of SB is about 2.5% better than SD in both areas. Hence, it can be stated that selecting the highest average SNR branch for SD does not always help in decoding the best signal because sometimes the same average SNR of two different signals lead to different outcomes because of their different instantaneous SNRs. Regardless of diversity technique used, it is clear that using base nodes in the ground network as a distributed antenna array for diversity combining improve the communication reliability. Moreover, the performance is analyzed here for a two-branch diversity system only and the improvement is still evident. In the final setup, we aim to use multiple base nodes for diversity combining and, hence, further improve the diversity gain.

## 4. System Verification

The current system has been thoroughly tested by tracking free-ranging bats of several species. During field deployments, researchers may adjust the complexity of the deployed system according to their research question, while either the full functionality (e.g., tracking, encounter detection, long-range telemetry for studying foraging behavior) may be used or only subsets (e.g., encounter detection and long-range telemetry for studying social behavior). These two scenarios have been tested in Forchheim, Germany, and Berlin, Germany, respectively, in the following referred to as “Forchheim field test” and “Berlin field test”.

### 4.1. Runtime

**Figure 23:**
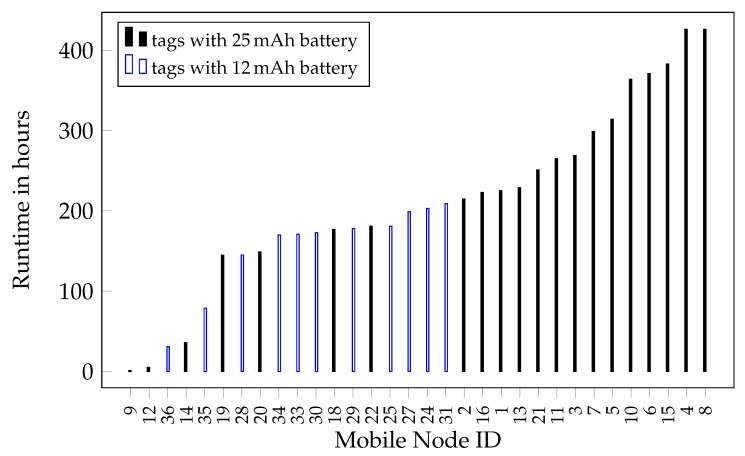
Runtime of each node in hours.

Differences in individual behavior causes significant variation in energy demand and battery life. Particularly when individuals spend considerable time within the ground station network, which leads to an increase in sampling rate, the power consumption is increased. The runtime of the mobile nodes deployed in the Berlin field test is shown in Figure 23. Here, batteries with 15 mAh, respectively, 25 mAh have been used according to the maximum acceptable weight for the corresponding individuals.

The runtime has been calculated from the first to the last radio contact to each node. This includes direct contact to a base station and also encounters between tagged animals. A low runtime can be explained by the corresponding animal leaving the covered area and thus preventing the mobile node to be received by any other sensor network node or base station. With small (12 mAh) batteries, a runtime of up to 209 h (nearly nine days) and with big (25 mAh) batteries a runtime of up to 426 h (nearly 18 days) has been recorded. On average, the sensor tag draws a current of 55 µA. This includes a low power sleep state with enabled wake-up receiver as well as periodic wake-ups to send out beacons every 2 s and check for base station contact to transmit the stored data. A slower beaconing frequency would lead to a reduced energy consumption but would also decrease the time resolution of recorded encounters and in turn increase the risk of missing short encounters. During the runtime seen in Figure 23, a total of 60,000 meetings have been recorded and 2.7 million received pseudo localization beacons have been recorded. These pseudo localization beacons are used to give a rough location of the animal based on which base station can receive the beacons.

### 4.2. Trajectories

**Figure 24:**
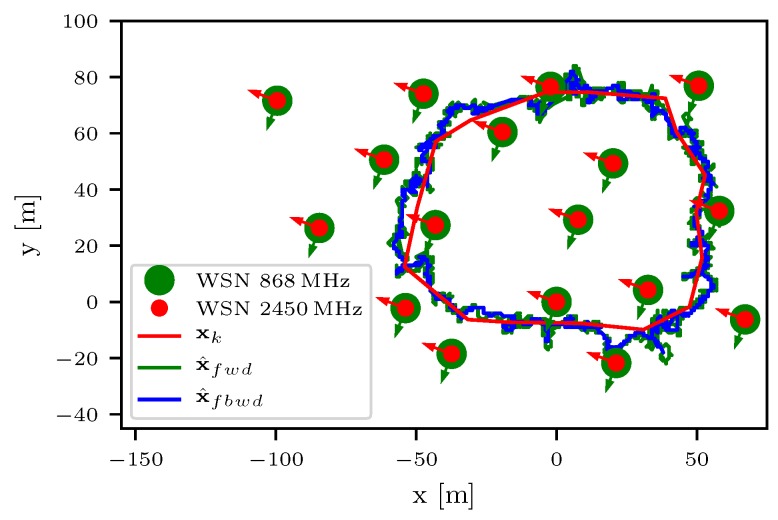
Localization sensor network with 17 nodes, operation at 868 MHz and 2.4 GHz. The ground true path $x_{k}$, the localized filtered path ${\hat{x}}_{fwd}$ and localized smoothed path ${\hat{x}}_{fbwd}$ is shown.

**Figure 25:**
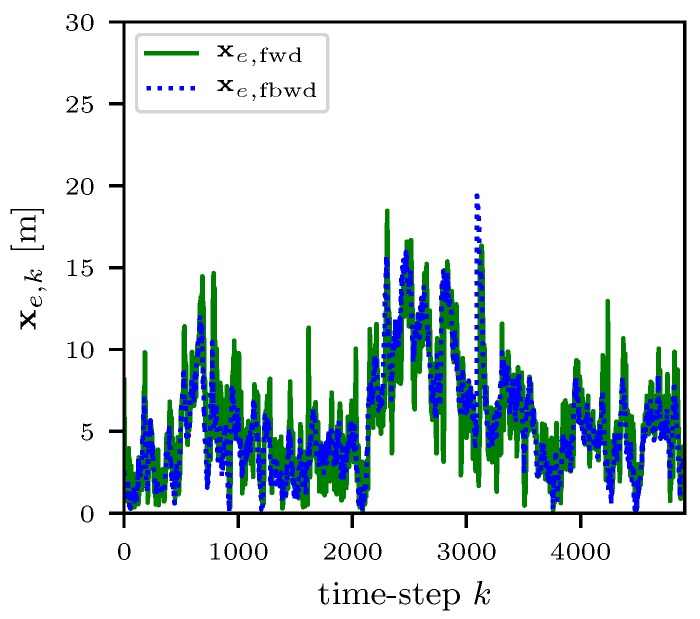
Localization error for all *k* time-steps of the filtered and smoothed estimated trajectory for the combination of the two frequencies 868 MHz and 2.4 GHz.

The localization methods presented in Section 3.3 were validated during the Forchheim field test in summer 2017. The localization network at the test site was operated for 18 days with 17 fixed sensor nodes. During the field-test, 14 bats were equipped with mobile sensor nodes. For the system performance verification, a reference path with 4912 waypoints is used. The reference path itself was measured by laser equipment during the daytime. In Figure 24, a sensor network with 17 fixed sensor nodes is shown. The red trajectory represents the ground truth $x_{k}$ of the reference path. The green and blue trajectory shows the filtered ${\hat{x}}_{fwd}$ and smoothed ${\hat{x}}_{fbwd}$ localized trajectory by the grid based particle filter presented in Ref. [56] for both frequencies. The localization error $x_{e,k}$ is calculated with

(8) $$x_{e,k}={\hat{x}}_{k}-x_{k},$$

where ${\hat{x}}_{k}$ represents the estimated position at the time-step *k* and shown in Figure 25. The average localization error is calculated by the mean Euclidean distance with

(9) $${\bar{x}}_{e}=\frac{1}{K}\sum_{k=0}^{K-1}{∥x_{e,k}∥}_{2},$$

where ${∥\cdot ∥}_{2}$ represents the Euclidean norm. In Table 1, the average localization error ${\bar{x}}_{e,k}$ for the different frequencies 868 MHz and 2.4 GHz is shown. Furthermore, the average localization error for the Maximum-Likelihood (ML) ${\bar{x}}_{e,\mathrm{ML}},$ filtered ${\bar{x}}_{e,\mathrm{fwd}}$ and smoothed ${\bar{x}}_{e,\mathrm{fbwd}}$ localization is shown. The diversity of the two frequencies lowers the localization error significant. It also shows that a adaptive localization performance can be achieved by using only one frequency if a lower accuracy is acceptable. By adding the second frequency, a more accurate localization is possible by the drawback of a higher energy consumption at the mobile node. For further energy saving, the transmit rate can be adapted from 1 Hz to 8 Hz.

### 4.3. Long Range Telemetry

**Figure 26:**
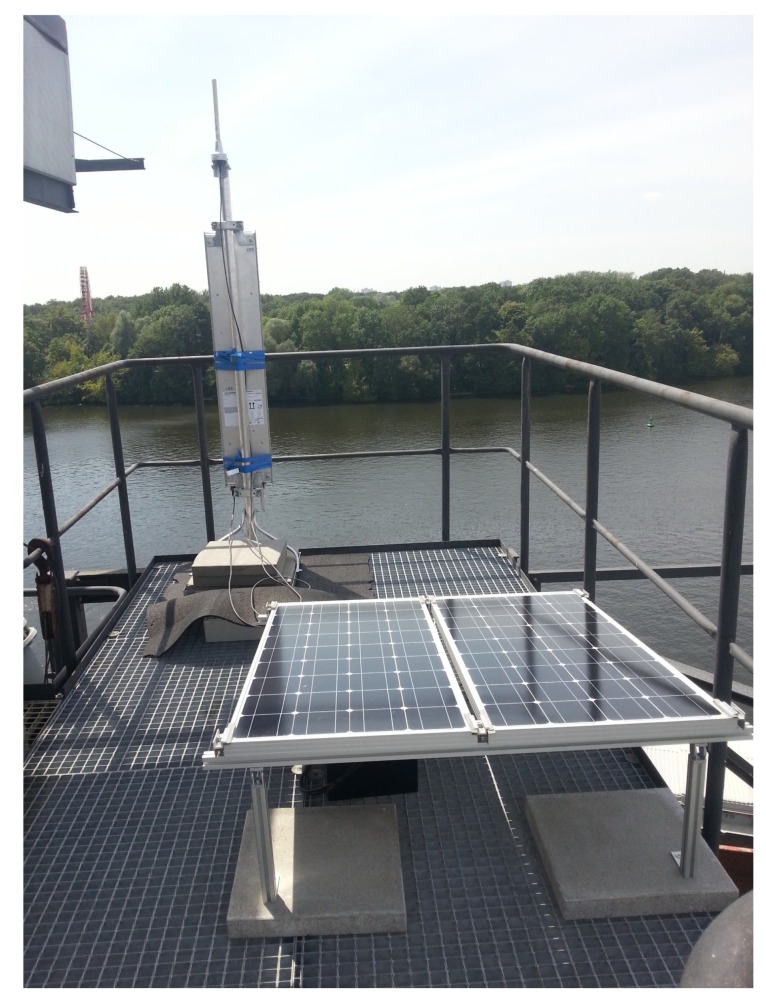
Long range telemetry base station.

The long range functionality as presented in Section 3.4 was validated during the Berlin field test in 2017. For this purpose, a total number of 32 individual bats were equipped with sensor nodes. A wake-up signal was issued every 2 s for encounter detection and telemetry transmission, utilizing the combined modulation as described before. Two long range base stations were supplied on exposed sites around the bats habitat at the forest of Treptower Park, Plänter Wald and Königsheide. Figure 26 shows a picture of one of the stations, with its directional antenna facing towards the forest, the omni-directional antenna on the top and the solar panel for autonomous operation (compare Section 3.4.2).

**Figure 27:**
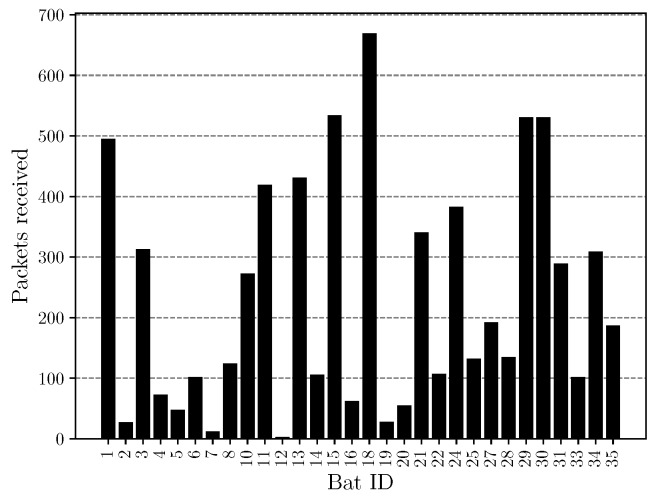
Received long range packets.

The field trial was carried out for two weeks. During this time, it was possible to receive over 7000 complete long range telemetry packets, accounting for over 168.000 bursts in total. Figure 27 shows the total number of received long range data packets in dependence of the bat identification number. As can be observed, we were able to receive packets from almost every captured bat. The base stations were located in distances of about 4 km around the forest, in heights comparable to those applied for the simulations performed in Refs. [43,44]. The successful receiving and decoding of data over this distance shows, that the system layout complies with our simulations on expected path loss, channel steadiness and rate. Based on those findings, the implementations also proved their functionality. For the measurements, the bat nodes transmitted a time stamp indicating their operation time as payload for the long range telemetry, as other data (see Section 3.4) was planned, but the sensors have not been equipped at that time. For the longest operation time, we encountered a value of 320 h of bat with ID 15, obtained by the very last contact to one of the long range base stations. When compared to the ground network in Figure 23 with 383 h of operation for ID 15, the long range transmission deems to deliver reasonable results, being a worthwhile extension to not only observe bats resting under the trees near the ground network, but also bats flying up in the air while hunting and outside the tracking range. In summary, we showed that we were able to assure a robust transmission even under the harsh constraints of a limited transmit power of just about 9dBm, strong path loss and shadowing of over 150 dB as well as unfavorable antenna alignment of the small bat node rod antenna relative to our receiving antennas. The measurements substantiated the simulation results and practical implementations.

## 5. Discussion

The BATS system for the first time combines two key features of animal logging, i.e., proximity sensing among animals and tracking of animals. Proximity information is processed and stored on the mobile nodes and transferred to ground stations upon contact. Tracking is aimed to achieve high spatial resolution at the cost of covering relatively small areas of usually a few hectares. Nevertheless, the overall aim is minimizing the weight of mobile nodes while still achieving a long runtime. Weight is the key parameter for the applicability of a technical animal logging system since low weight allows for tagging of smaller animals. Minimizing weight comes at the cost of reduced runtime and/or functionality. Therefore, during system development, we always aimed to allow flexibility in hardware (e.g., battery size, number of sensors on a mobile mode) and software settings in order to allow adaptations of the BATS system to specific applications.

A direct comparison of individual parts of the BATS project to other systems is difficult for multiple reasons. Each part of the project is highly optimized to perform in perfect harmony with the other components. This way, the full performance of each sub-project only takes effect when embedded in the whole system. This is particularly the case because the ultra low power goal of the overall system poses a strong limitation in system design. This makes the sub-projects not directly comparable to other system that have full control over the whole experimental setup. Detailed literature about the performance of individual components of other tracking systems, such as those mentioned in the related work Section 2, is rare. Therefore, only the performance of the system as a whole can be compared. Table 2 gives an overview about selected related system and compares key parameters of the systems with each other. Afterwards, the systems are compared in more detail.

For encounter detection, the Encounternet project (see Section 2) has similar goals. However, as seen in Figure 23, the mobile node runtime of the nodes developed in the BATS project is surpassing the Encounternet node runtime of 21 h (7.5 days in transmit only mode making them visible for other nodes/base stations, but they can’t record meetings themselves) while having the same weight of around 1.3 g. The BATS system achieves up to 420 h in full duplex encounter detection. This is mostly possible because of the wake up receiver based approach of the BATS project. This way, the main receiver and the whole circuit can stay in a low power state most of the time. Other factors benefiting the long runtime of the BATS sensor nodes is smart software scheduling and the selection of energy efficient components even if this means a slightly degraded performance. Other than Encounternet, the BATS project also allows precise location tracking in a predefined ground node network.

The tracking part of the BATS system can best be compared to ATLAS, MOTUS and ICARUS as described in Section 2. However, these systems and the BATS project serve different purpose in animal research. While the former are focusing on the large scale tracking to for example research migration patterns, BATS focuses tracking bats with high spatial resolution in a small area of a few hectares.

Regarding spatial resolution, the ATLAS system and GPS based systems like ICARUS can be compared to BATS as long as the tracking is performed under ideal conditions. However, based on the system architecture, the ATLAS system reacts sensitively to multipath propagation, which has a negative effect on the precision of the localization. In Section 3.3.2, we described how the BATS system reduces such negative impact due to a new multipath robust design. This allows precise localization results even in multipath propagation affected areas like forests. While the performance of GPS based systems can be seen as relatively precise in open areas, the precision is reduced in areas with diminished reception such as vegetated areas. In particular, light low power GPS sensor tags may suffer a drop in performance under such conditions. Since in the BATS system the ground stations are deployed directly in the area that is most relevant to the research, the base station grid can be planned in a way that coverage is optimized in this area of interest. However, this of course limits the size of the covered area while GPS can be used global and without ground infrastructure.

In particular, ATLAS and MOTUS make use of relatively simple sensor nodes with the data processing mostly on the ground stations. Energy efficiency isn’t that critical there anymore. This results in relatively long mobile node runtime due to its simplicity. In ICARUS, the localization is performed via GPS fixes on the node. Increasing the energy demand drastically but also makes the sensor nodes independent of a ground station network. ICARUS uses mobile nodes with attached solar panels. This way, a long runtime can be achieved despite the relatively high current consumption of GPS trackers. This approach can not be applied in the BATS projects since the investigated animals, bats, are nocturnal species and hide in dark roost during day. Still, the theoretically unlimited runtime of the ICARUS nodes is a desirable characteristics of the node. Further research is required to design an energy harvesting system to support the BATS mobile node while taking the strict weight limit as well as the fact that bats are nocturnal animals into account.

Similar to ATLAS and MOTUS, the BATS project offloads energy-intensive localization to the less energy-constrained ground station network but keeps the mobile nodes highly functional. The localization of an animal is approximated according to the recording of mobile node beacons received by ground nodes that are processed by a central computer proving almost real time information of an animal’s location. However, due to the capabilities of the mobile node, we can limit sending location beacons only to the area where mobile base stations are in range. This way, the overall energy consumption can be reduced especially for individuals spending only short periods of time in the tracking grid.

The combination of encounter detection and ground system based localization drastically reduces the amount of so called “blind spots” where no information on the animal can be obtained. GPS based systems for example may have problems getting GPS fixes that determine the current location in thick forest environments and places like roosts or caves. In unknown locations, the BATS system still allows for indirectly monitoring the animals’ behavior by collecting encounter data among tagged animals or—if such locations are known—fixed sensor nodes may be installed in these places that act as normal bat nodes. This way, fixed nodes record encounter data and report on the identity of individual tagged bats in range. In such scenarios, we can precisely assess, e.g., the time an individual left the roost for hunting.

Other than all mentioned systems, the BATS system implements a quasi energy neutral long range telemetry system that allows for receiving data even if the bat leaves the area of high interest where the precise tracking and/or data download take place. Thanks to embedding, the long range telemetry signal in the beacons sent out for encounter detection and making use of a newly developed transmission scheme (as described in Section 3.4.1), the long range telemetry is included at practically no extra current consumption on the mobile node. We achieve a high energy efficiency and still can receive the signal up to 4 km away from the long range antennas despite high path loss, shadowing and generally highly variating channels due to the bats’ movement speed.

Being able to adapt its behavior based on the current situation to increase energy-efficiency while maintaining full functionality when needed is the key feature of the BATS system. Various functions on the mobile node are automatically regulated upon demand by switching between different operation levels including a sleep mode. Such adaptive functionality is achieved by introducing so-called zones that are set by receiving beacons from ground stations. Similar to only sending localization beacons at high frequency while being in the tracking grid and not sending them while being outside the grid to save energy, for encounter logging, the beaconing frequency is reduced as soon as the animal is within the roost. Here, the environment is rather stable, thus the time between encounter beacons is much longer. Following this zone principle, certain functionalities can be enabled respectively disabled and parameters of the tags can be changed depending on the current application of the system. This functionality enables the BATS system to track bats inside the tracking grid at high spatial and temporal resolution while no localization beacons are sent at all when bats are outside the tracking grid to save energy. Comparable systems either transmit beacons continuously or schedule the sending based on programmed time slots regardless of the location of the animal. Apart from external control of the nodes behavior with beacons, the mobile node can schedule the use of on board peripheral resources, like the NVRAM storage, itself. This allows the system to keep components turned off for the majority of the time and only power them when required.

A particularly energy intensive task of the mobile node is the transmission of stored data to the ground station network. Combining multiple packages to bursts allowed for the reduction of energy by 40% to 30 µJ per packet. Further reductions may be possible by omitting checksums in the transmitted packages. However, this would lead to an increased packet loss and render the recorded data unusable.

Data retrieval from mobile nodes is a crucial step of the BATS system. It occurs upon contact of mobile nodes to ground stations at a distance of up to approximately 150 m depending on the environment. Much longer data retrieval distances of up to 4 km were achieved by the implementation of long-range telemetry (see Section 3.4).

Technical development of the BATS system was subjected to multiple iterations for optimization and addition of new features. An overview about a previous version can be found in Ref. [58]. Meanwhile, it is possible to track 60 animals at the same time instead of 28 previously. The spatial precision of the flight trajectory was substantially improved from 7 m average error to 4 m. In addition, the current mobile node is characterized by lighter hardware and slimmer dimensions due to improved hardware components and a redesign of the board outlay. Low energy-consumption was an important criterion in selecting among commercially available components for the mobile nodes. Thanks to the now available on board NVRAM, the system also allows recording of larger data sets (e.g., more encounters) until download upon contact to a ground station. The lower weight of the node without a battery and lower power consumption allows for the use of batteries with higher capacity and generally results in an extended runtime.

## 6. Conclusions and Future Work

The wide range of available technical systems for animal logging can largely differ in their technical features determining their applicability studying animals in their natural habitat Section 2. The BATS system simultaneously meets the needs for proximity sensing and local high-resolution tracking in a single system by optimizing energy use. This unique combination allows a wide range of applications in the fields of sociobiology, behavioral ecology, movement ecology or physiological ecology. The small size and weight of the mobile nodes and the flexibility of the whole system allow for investigating a broad spectrum of species.

Depending on the actual use case, individual functionalities of the system can be disabled to provide a longer runtime and certain functions can flexibly be enabled/disabled in the field based on the current location/situation of the tag. The adaptive approach of the BATS system leads to high quality data when required while at the same time maintaining an overall long runtime.

The current system is actively used in biological studies [59,60] and creates rich data sets on the studied animals.

The applicability of the BATS system can be expanded by adding new sensors like an accelerometer or magnetometer. Thus, it represents a new modular system that can be redesigned according to the needs for a specific application. Both hardware and software adjustments are important measures to reach the minimal weight limits of mobile nodes, which strongly determines the range of animal species that can be studied.

However, even the current version can be used for multiple further biological studies regarding (social) behavior in bats and similar sized (airborne) animals. The paper has shown that, for studies that rely on encounter data either with or without localization, the BATS system has outstanding performance and is capable of generating precise results.

## Figures and Tables

**Table 1:** Average localization error ${\bar{x}}_{e}$ of the Maximum-Likelihood estimator ML, filtered and smoothed estimated trajectory for the separate frequencies and the combined results.

Method	*f* [MHz]	${\bar{x}}_{e,\mathrm{ML}}$ [m]	${\bar{x}}_{e,\mathrm{fwd}}$ [m]	${\bar{x}}_{e,\mathrm{fbwd}}$ [m]
Δ*RSSI*	868	36.33	7.72	7.02
Δ*RSSI*	2450	14.64	6.42	6.28
Δ*RSSI*	868, 2450	12.93	5.58	5.47

**Table 2:** Comparison of related bat tracking systems.

System	Weight	Runtime	Spatial Resolution	Coverage	Proximity Detection
ATLAS [12]	1.5 g	10 days	5 m	several km${}^{2}$	no
MOTUS [13]	0.2 to 2.6 g	10 days to 3 years	500 m to 15 km	multiple thousand km${}^{2}$	no
Biotrack PinPoint 10 [57] (GPS Tracker)	1 g	up to 130 fixes	few meters (GPS)	global	no
ICARUS [16]	5 g	theoretically unlimited because of solar panels	few meters (GPS)	global	no
Encounternet [14]	1.3 g (10 g)	21 hours (2 month)	presence detection	N/A	yes
BATS (this work)	1.0 g (1.3 g)	8 days (17 days)	4 m	several hectares	yes (around 5 m)
